# *In Planta* Biocontrol of *Pectobacterium atrosepticum* by *Rhodococcus erythropolis* Involves Silencing of Pathogen Communication by the Rhodococcal Gamma-Lactone Catabolic Pathway

**DOI:** 10.1371/journal.pone.0066642

**Published:** 2013-06-21

**Authors:** Corinne Barbey, Alexandre Crépin, Dorian Bergeau, Asma Ouchiha, Lily Mijouin, Laure Taupin, Nicole Orange, Marc Feuilloley, Alain Dufour, Jean-François Burini, Xavier Latour

**Affiliations:** 1 Laboratoire de Microbiologie Signaux et Microenvironnement (LMSM EA 4312) - Normandie Université - Université de Rouen - IUT Evreux, Evreux, France; 2 SIPRE Comité Nord Stations de Recherche et de Création Variétale, Bretteville du Grand Caux et Achicourt, France; 3 Laboratoire de Biotechnologie et Chimie Marines - EA 3884 - Université de Bretagne-Sud, IUEM, Lorient, France; The Scripps Research Institute and Sorrento Therapeutics, Inc., United States of America

## Abstract

The virulence of numerous Gram-negative bacteria is under the control of a quorum sensing process based on synthesis and perception of *N*-acyl homoserine lactones. *Rhodococcus erythropolis*, a Gram-positive bacterium, has recently been proposed as a biocontrol agent for plant protection against soft-rot bacteria, including *Pectobacterium*. Here, we show that the γ-lactone catabolic pathway of *R. erythropolis* disrupts *Pectobacterium* communication and prevents plant soft-rot. We report the first characterization and demonstration of *N*-acyl homoserine lactone quenching *in planta*. In particular, we describe the transcription of the *R. erythropolis* lactonase gene, encoding the key enzyme of this pathway, and the subsequent lactone breakdown. The role of this catabolic pathway in biocontrol activity was confirmed by deletion of the lactonase gene from *R. erythropolis* and also its heterologous expression in *Escherichia coli*. The γ-lactone catabolic pathway is induced by pathogen communication rather than by pathogen invasion. This is thus a novel and unusual biocontrol pathway, differing from those previously described as protecting plants from phytopathogens. These findings also suggest the existence of an additional pathway contributing to plant protection.

## Introduction

Numerous Gram-negative plant-pathogenic bacteria use a communication system based on synthesis and perception of *N*-acyl-homoserine lactones (NAHSLs); this system involves a quorum sensing (QS) mechanism that controls genes determining pathogenicity and colonization of the host surface [1]. The QS mechanisms of the causative agents of soft-rot, including members of the genera *Erwinia* now reclassified as *Dickeya* and *Pectobacterium* [2]–[4], have been extensively studied. They have also been documented in the economically important phytopathogen *Pectobacterium atrosepticum* under host influence [5]–[7]. QS triggers an attack by soft-rot bacteria once there is a sufficient number of bacterial cells, and ensures the coordinated production of massive amounts of lytic enzymes to degrade plant tissues and thereby obtain nutrients [8]. This coordinated overproduction of lytic enzymes is suspected to help soft-rot bacteria overwhelm plant defenses [9]. Generating signaling molecules is therefore an important element of the plant-pathogen interaction. Liu and co-authors [10] estimated that transcription of 26% of the genes in *P. atrosepticum* SCRI1043, including numerous virulence genes, differed between a NAHSL synthase mutant and the wild-type, further implicating QS in pathogenesis. These observations suggest that the means of communication used by pathogenic bacteria are potential targets for the development of a novel biocontrol strategy: rather than directly eradicating the pathogen, interfering with communication may reduce the expression of virulence systems and thus pathogenicity [11]–[13].The potato is one of the world’s major crops [14], [15] and epidemiologic studies report devastating potato diseases suggestive of the emergence of new pectinolytic agents [16], [17]. We are therefore developing a novel biocontrol strategy for the *S. tuberosum* model based on the selective stimulation of NAHSL-degrading bacteria [18]–[20]. This approach to soft-rot control involves the use of the Gram-positive *Rhodococcus erythropolis* strain R138 as a biocontrol agent (BCA): it is able both to degrade diverse γ-lactones effectively *in vitro* [21] and to suppress the maceration of tubers in hydroponic and field culture conditions [22], [23].

However, the mechanism by which this strain controls soft-rot has never been elucidated *in planta*, although it is suspected that it is linked to NAHSL-breakdown. We recently discovered a lactone assimilation pathway in *R. erythropolis*: the lactonase QsdA hydrolyzes the lactone bond of a wide range of flavoring compounds containing a γ-butyrolactone ring coupled to an alkyl chain; the open-chain form of these substrates is then degraded by a β- or ω-oxidation step and the products join the β-ketoadipate pathway and the Krebs cycle [21]. Here, we report an analysis of the involvement of this γ-lactone catabolic pathway in the control of tuber soft-rot by *R. erythropolis* R138. The functions of QsdA, the key enzyme of this catabolic pathway, were investigated by transferring the *qsdA* gene to a heterologous host (*Escherichia coli*). Also, we constructed a *R. erythropolis* R138 *qsdA* deletion mutant and studied NAHSL breakdown in tubers in its presence. *In situ qsdA* transcription was followed by confocal laser scanning microscopy (CLSM) using an *R. erythropolis* strain carrying a plasmid-borne *qsdA::gfp* transcriptional fusion. The QS process and tuber soft-rot were generated using a virulent *P. atrosepticum* isolate and were quenched with a recombinant of this strain synthesizing an NAHSL-lactonase and consequently producing only negligible amounts of NAHSLs. *Pectobacterium* quorum sensing and rhodococcal quorum quenching were successfully characterized *in planta*. We demonstrate that the γ-lactone catabolic pathway is induced by pathogen communication, and is involved both in NAHSL breakdown and in the control of the disease.

## Materials and Methods

### Bacterial Strains, Growth Media and Culture Conditions

The characteristics of bacterial strains and plasmids used are presented in Table 1. *Agrobacterium tumefaciens* NT1 used as a biosensor to detect NAHSL was grown in minimal *Agrobacterium* Broth (AB) at 25°C [24]. *Pectobacterium atrosepticum* CFBP 6276 was grown in PGA minimal medium supplemented with 0.4% (w/v) polygalacturonic acid (Sigma-Aldrich, St. Louis) at 25°C as described elsewhere [25] and *Escherichia coli* DH5α in Luria-Bertani medium (AES Chemunex, Bruz, France) at 37°C. For *R. erythropolis* strains cultivated at 25°C, the non-selective growth media used were TY to prepare bacterial suspensions for inoculation of potato tubers and LBP for the conjugative transfer of DNA. TY medium contained 0.5% (w/v) tryptone (Difco, Le Pont de Claix, France), 0.2% (w/v) yeast extract (Difco) and 5% (v/v) of a phosphate buffer (6% (w/v) K_2_HPO_4_ (Merck, Fontenay-sous-Bois, France) and 4% (w/v) NaH_2_PO_4_ (Merck)). LBP medium contained 1% (w/v) Bacto Peptone (Difco), 0.5% (wt/v) yeast extract, and 1% (w/v) NaCl (Sigma-Aldrich). To evaluate *R. erythropolis* R138 density in potato tubers, the minimal medium described by Barbey et al. [21] was used, with the following modification: gamma-caprolactone (GCL, Sigma-Aldrich), the sole carbon source, was included at 2 g/l. Growth media were supplemented as appropriate with kanamycin at a concentration of 30 µg/ml for *E. coli* and 200 µg/ml for *R. erythropolis*, and with 10 µg/ml tetracycline for *P. atrosepticum*, or 100 µg/ml ampicillin for *E. coli*, and solidified with agar (15 g/l). All cultures were grown on a rotary shaker (180 rpm). Growth was monitored spectrophotometrically at 580 nm. All cultures were inoculated at an initial OD_580_ of 0.05.

**Table 1:** Bacterial strains and plasmids.

Strain or plasmid (synonym)	Relevant characteristic(s)	Source or reference
***Agrobacterium tumefaciens***		
*A. tumefaciens* NT1	NT1 derivative of strain C58, carrying pZLR4; Biosensor usedfor NAHSL detection; Gm^R^	[38]
***Escherichia coli***		
S17-1	recA pro hsdR RP4-2-Tc::Mu-Km::Tn*7*	[49]
DH5α	Host for cloning; SupE44 ΔlacU169 (Φ80lacZΔM15) hsdR17recA1 endA1 gyrA96 thi-1 relA1	Lab collection
DH5α(pUC19) (*Ec*)	Strain DH5α carrying pUC19; Ap^R^	This study
DH5α(pUC19-*qsdA*) (*Ec-qsdA*)	QsdA-expressing DH5α; Ap^R^	This study
***Pectobacterium atrosepticum***		
CFBP 6276	Potato soft-rot pathogen; NAHSL producer	[25]
6276(pME6000) (*Pa*-QS+)	Strain 6276 carrying pME6000, a broad-host-range cloningvector; Tc^R^	[40]
6276(pME6000-*aiiA*) (*Pa*-QS**–**)	Strain 6276 carrying pME6000 containing the *aiiA* genefrom *Bacillus* sp. A24 under the constitutive P_lac_ promoter; Tc^R^	[40]
***Rhodococcus erythropolis***		
R138 (BCA)	NAHSL-degrading isolate obtained from hydroponic cultureof potato plants	[50]
R138(pEPR1*-qsdA*::*gfp*) (BCA*-qsdA*::*gfp*)	R138 transformed with pEPR1 *qsdA*-*gfp*	This study
R138 Δ*qsdA* (BCA*-*Δ*qsdA*)	R138 with a 813 bp fragment deleted from the *qsdA* gene	This study
**Plasmids**		
pZLR4	*traG::lacZ/traR* reporter system	[38]
pME6000	Broad-host-range cloning vector, Tc^R^	[51]
pME6000-*aiiA* (pME6863)	pME6000 carrying the *aiiA* gene from *Bacillus* sp. A24 underthe constitutive P*_lac_* promoter	[52]
pAKE604	Conjugative suicide vector for *qsdA* gene deletion; Km^R^	[26]
pAKE604 Δ*qsdA*	pAKE604 containing the *qsdA* upstream and downstreamregions; Km^R^	This study
pEPR1	Shuttle promoter-probe vector carrying the promoterlessgfp_uv_ reporter gene; Km^R^	[28]
pEPR1-*qsdA*::*gfp*	pEPR1 with a *qsdA*::*gfp*_uv_ transcriptional fusion; Km^R^	This study
pUC19	Cloning vector for *E. coli*; Ap^R^	Lab. collection
pUC19-*qsdA*	pUC19 with a 1376 bp PCR fragment containing the *qsdA*gene; Ap^R^	This study

Km^R^, Ap^R^, Gm^R^ and Tc^R^ indicate resistance to kanamycin, ampicillin, gentamicin and tetracycline, respectively. NAHSL, *N*-acyl homoserine lactone; CFBP, Collection Française de Bactéries associées aux Plantes, Institut National de la Recherche Agronomique (INRA), Angers, France.

### Construction of a*qsdA* Deletion Mutant of *R. erythropolis* R138

A markerless *qsdA* deletion mutant was constructed by using the conjugative suicide vector pAKE604 [26], which cannot replicate in *R. erythropolis*. The regions upstream and downstream (919 and 780 bp) from *qsdA* were amplified using standard conditions with Extensor Hi Fidelity polymerase (Thermo scientific, Courtaboeuf, France) and the oligonucleotide pairs qsdA_up_fw/qsdA_up_rv (5′ GTATG*TCTAGA*TGAATCCTGTGTGGTCGTC 3′ with the *Xba*I site italicized; 5′ TGAAC*ATGCAT*GACATGCTCGTGCATCAAC 3′ with the *Nsi*I site italicized) and qsdA_do_fw/qsdA_do_rv (5′ CTAGT*ATGCAT*CTGCTCGAACGTGGTGTCA 3′ with the *Nsi*I site italicized; 5′ CAGAT*GAATTC*GATAGATCTGCGCTGCG 3′ with the *EcoR*I site italicized), respectively. These primers were designed from the sequence of the relevant 5 kb of the genome of *R. erythropolis* R138, kindly provided by D. Faure (CNRS, ISV Gif/Yvette, France). The PCR fragments were inserted into pAKE604 to give pAKE604 Δ*qsdA* which was transferred into *R. erythropolis* R138 by biparental mating, as previously described by van der Geize et al. [27] with the following modifications: donor and recipient cells were mixed and spotted onto sterile nitrocellulose filters (GE Healthcare, Orsay, France), which were placed on dry LBP agar plates overnight at 30°C. Then, the mating mixture was suspended in 1 ml LBP liquid medium and 0.1 ml aliquots were spread on LBP agar plates supplemented with kanamycin (200 µg/ml), to select for the presence of the plasmid, and nalidixic acid (30 µg/ml) to kill the *E. coli* S17-1 donor cells. To test for the excision of the suicide plasmid from the chromosomal DNA, the co-integrate isolate was suspended in 1 ml of sterile saline solution and 0.1 ml aliquots of serial dilutions were plated on LBP agar medium supplemented with 10% sucrose. PCR analysis then DNA sequencing were used to verify the construction of the in-frame *qsdA* deletion and resulting truncated version of the *qsdA* gene (156 bp in length instead of 969 bp for the wild-type gene).

### Construction of a*qsdA::gfp* Transcriptional Fusion in *R.* *erythropolis* R138 Strain

The promoter region of the *qsdA* gene was amplified with the Extensor Hi Fidelity polymerase using primers FQDAN (5′ CCA*ATGCAT*GGTAGGCATCGGGACATTCT 3′ with *Nsi*I site italicized) and RQDAB (5′ CGC*GGATCC*CGATCGAACCCCTGACTGT 3′ with *Bam*HI site italicized) and cloned as a 184-bp *Nsi*I-*Bam*HI fragment into the vector pEPR1 [28] upstream from the *gfp* gene, creating a *qsdA*::*gfp* transcriptional fusion. After sequencing the insert, this plasmid-designated pEPR1-*qsdA*::*gfp* was introduced in *R. erythropolis* R138 cells by electroporation as described by Vesely et al. [29]. Briefly, 100 µl of electrocompetent bacterial cells were mixed with 0.5 µg of plasmid DNA and electroshocked at 2.5 kV for 7 ms using a Savant electroporator (Thermo Fisher Scientific). After electroporation, bacterial cells were cultivated in 1 ml of TY broth at 25°C for 5 h with shaking (180 rpm) then plated on TY medium containing kanamycin and incubated for 3 days at 25°C. Among the Km^r^ clones obtained, one was chosen and checked for the presence of pEPR1*qsdA*-*gfp* by PCR analysis.

### Heterologous Expression of QsdA in*E. coli*

The *qsdA* gene including its promoter region was amplified using primers QsdAF (5′TAATAA*GAATTC*TGACATGTCGAGTGGTTCCT 3′ with *EcoR*I site italicized) and QsdAR (5′TAATAA*TCTAGA*GCTGACAGTCCTGTCGAAGT 3′ with *Xba*I site italicized) and the amplified fragment was inserted into pUC19 to give pUC19-*qsdA*; this was introduced into *E. coli* DH5α by transformation using the standard heat-shock method (42°C for 45 s). The appropriate insert was confirmed by sequencing.

### Biocontrol Assays on Potato Tubers

Overnight cultures of *P. atrosepticum* CFBP 6276, *R. erythropolis* R138, *E. coli* DH5α and their derivative strains (see Table 1) were washed in 0.9% NaCl. The surface of *Solanum tuberosum* cv. Allians tubers were sterilized by incubation in a 1% (v/v) bleach solution for 10 min followed by rinsing with distilled water. They were inoculated by injection into the intramedulla (to a depth of 1 cm) with 10 µl of cell suspension containing the following bacterial combinations: [10^7^ CFU of *P. atrosepticum* 6276/6276(pME6000)/6276(pME6000-*aiiA*) with 4×10^7^ CFU of *R. erythropolis* R138/R138 Δ*qsdA*/R138(pEPR1-*qsdA::gfp*)] or [10^7^ CFU of *P. atrosepticum* 6276 with 2×10^7^ CFU of *E. coli* DH5α(pUC19)/DH5α(pUC19-*qsdA*)]. For controls, the suspensions of *R. erythropolis* R138 and/or *P. atrosepticum* 6276 were replaced with 0.9% NaCl. The inoculated tubers were incubated in a Minitron incubator (Infors, Massy, France) at 25°C with a relative humidity of 80±2%. One, two, three and seven days after infection, eight to 18 tubers for each condition were sectioned across the middle, and photographed. These experiments were conducted three times at different times of the year. The Mann and Whitney test was used to assess differences in maceration symptoms between groups (*α* = 0.05).

### Analysis of Bacterial Populations Collected from Inoculated Potato Tubers

A 1 cm-diameter cookie cutter was used to collect standardized tuber samples from both parts of each sectioned tuber. Each sample was homogenized and 500 mg aliquots of the tuber homogenate were suspended in 5 ml of sterile saline solution. The suspensions were serially diluted, and plated on PGA minimal medium, selective minimal medium containing GCL, TY medium supplemented with 200 µg/ml kanamycin, and LB supplemented with 100 µg/ml ampicillin to count *P. atrosepticum* 6276, *R. erythropolis* R138, *R. erythropolis* R138(pEPR1-*qsdA*::*gfp*), and *E. coli* DH5α, respectively. Three independent experiments were performed and for each experiment, three independent samples were analyzed for each condition.

### Extraction and Quantification of NAHSLs from Potato Tubers

For each condition, NAHSL was extracted from three samples of three different potato tubers. Reported values are means ± SD of three independent experiments. Samples of 500 mg of tuber (fresh weight) were suspended in 2 ml of sterile saline solution and vortexed vigorously for 1 min. One ml of this suspension was then mixed with an equal volume of dichloromethane (Fisher scientific, Illkirch, France) and centrifuged at 3,000 g for 10 min. The organic phase was collected and the aqueous phase was again extracted with dichloromethane as described above. The two resulting organic phases were combined, dried over anhydrous magnesium sulfate (Sigma-Aldrich), evaporated to dryness, and stored at −20°C until analysis. These dried extracts were analyzed by HPLC-MS, as previously described [30], [31]. NAHSL was also assayed using the *A. tumefaciens* biosensor NT1 and thin-layer chromatography silicate plates (C18-reverse phase, Whatman) according to Shaw et al. [32]. Eleven NAHSL standards were used, six *N*-acyl- (from butanoyl- to tetradecanoyl-) and five *N*-3-oxo- (from 3-oxo-hexanoyl- to 3-oxo-tetradecanoyl-) -L-HSL. The synthetic standards and stock solutions were prepared in HPLC-grade ethyl acetate (Fisher Scientific, France) and stored at −20°C.

### Sampling of Potato Tubers for Confocal Laser Scanning Microscopy (CLSM)

Bacterial smears were made from inoculated potato tuber tissue and fixed by heating on glass slides. Slides were examined by inverted CLSM (LSM 710, Carl Zeiss MicroImaging, Le Pecq, France). To excite the GFP in bacterial cells, a 488 nm laser with 509 nm emission filters was used. Confocal images were acquired with Zen 2009® software (Carl Zeiss MicroImaging) using the same gains and offset parameters for all images. Three bacterial smears from three different tubers were analyzed for each condition.

## Results

### Tuber Soft-rot Associated with*P. atrosepticum* is Triggered by *N*-3-oxo-octanoyl-L-HSL-based QS

Two methods were tested for evaluating NAHSL production: high-performance liquid chromatography coupled to mass spectrometry (HPLC-MS) as described in Latour et al. [30] and thin-layer chromatography (TLC) with the biosensor *Agrobacterium tumefaciens* NT1 for detection [32]. TLC gave better results with all inoculated tubers, probably because it has a lower limit of NAHSL detection (0.5 fmol for *N*-3-oxo-octanoyl-L-HSL) [32]. This high sensitivity is a consequence of the signaling molecule produced by *P. atrosepticum* being the same as that used naturally by the biosensor *A. tumefaciens* and therefore being best recognized by this bacterium.

*In vitro* characterization of NAHSL production both by TLC and HPLC-MS showed that most *P. atrosepticum* strains, including the *P. atrosepticum* strain 6276 used in this study, produce *N*-3-oxo-octanoyl-L-HSL when grown in minimal medium with polygalacturonic acid [18]. Potato tubers were inoculated with *P. atrosepticum* 6276 and NAHSL was subsequently extracted. TLC identified the extracted NAHSL as *N*-3-oxo-octanoyl-L-HSL, the same as the NAHSLs produced by *P. atrosepticum* in a synthetic medium inducing synthesis of virulence factors [7], [18] and the signaling molecule used by this pathogen in the host plant. No NAHSLs were detected in tubers inoculated with *P. atrosepticum* 6276(pME6000-*aiiA*) expressing the *Bacillus* AiiA lactonase [33]. Hereafter, for simplicity, this NAHSL auto-quencher strain is referred to as *Pa*-QS–.

**Figure 1:**
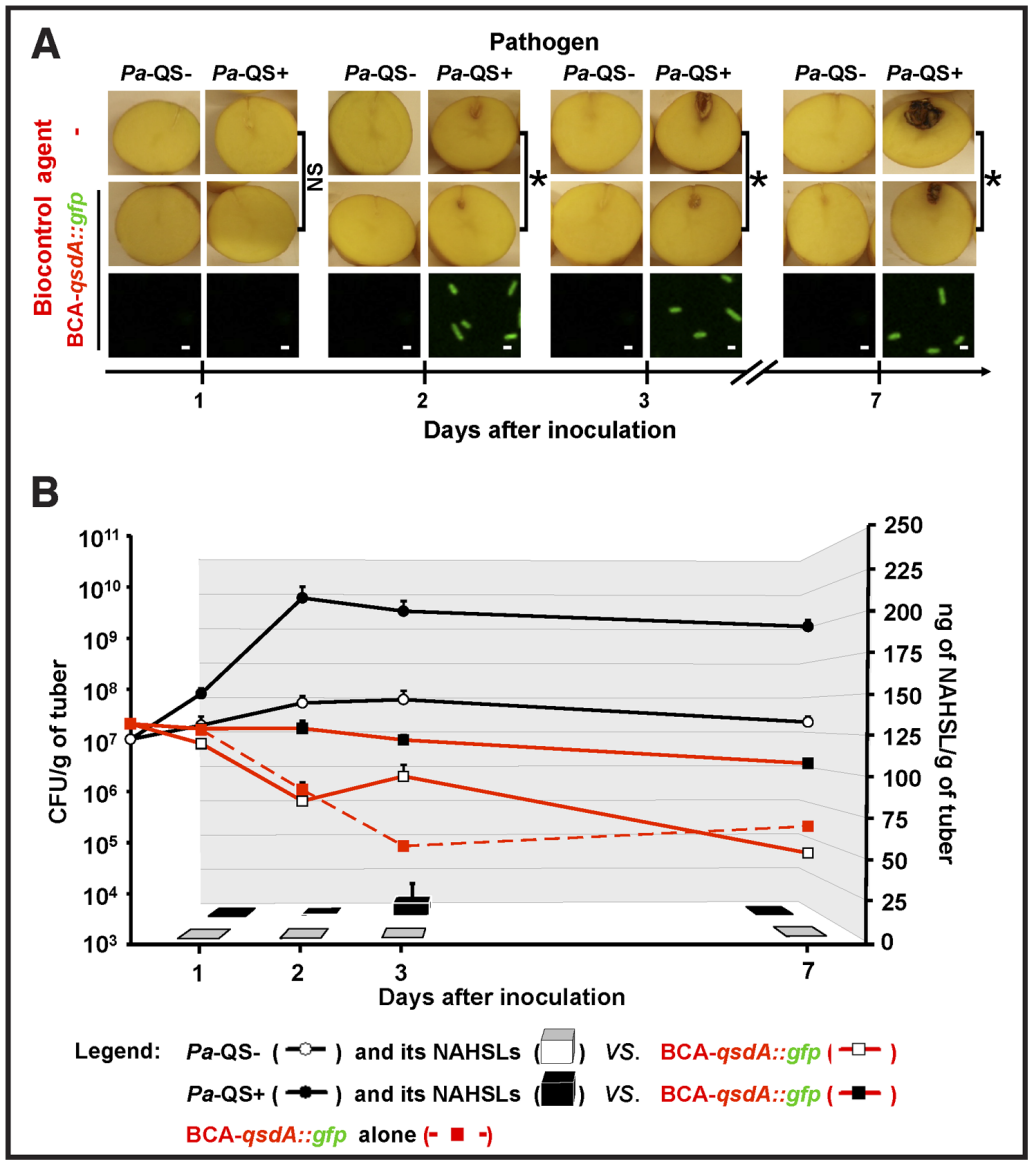
Induction of*qsdA* gene transcription, NAHSL-breakdown and biocontrol activity of ***R. erythropolis*** in potato tubers. (A) *qsdA* gene transcription and biocontrol activity of the *R. erythropolis* BCA-*qsdA::gfp* against *P. atrosepticum* 6276 defective (*Pa*-QS**–**) or not (*Pa*-QS**+**) for NAHSL production were analyzed at 1, 2, 3 and 7 days after inoculation of *S. tuberosum* var. Allians tubers. For the controls, one of the two strains was replaced in the inoculum with a 0.9% NaCl solution. Asterisks indicate significantly less severe maceration symptoms in the presence of the BCA-*qsdA::gfp*, as assessed with the Mann and Whitney test (*α* = 0.05). The fluorescence of the BCA-*qsdA::gfp* was analyzed by confocal laser scanning microscopy. (B) The numbers of *P. atrosepticum* (black lines) and *R. erythropolis* (red lines) bacteria per unit weight (CFU/g fresh weight of potato tubers), and NAHSL concentration (ng/g of potato tubers; black and white bars) were determined for each condition in potato tubers. For lines and bars, each value is the mean of three replicates with the standard deviation indicated. NS, non-significant; NAHSL, *N*-acyl homoserine lactone.

**Figure 2:**
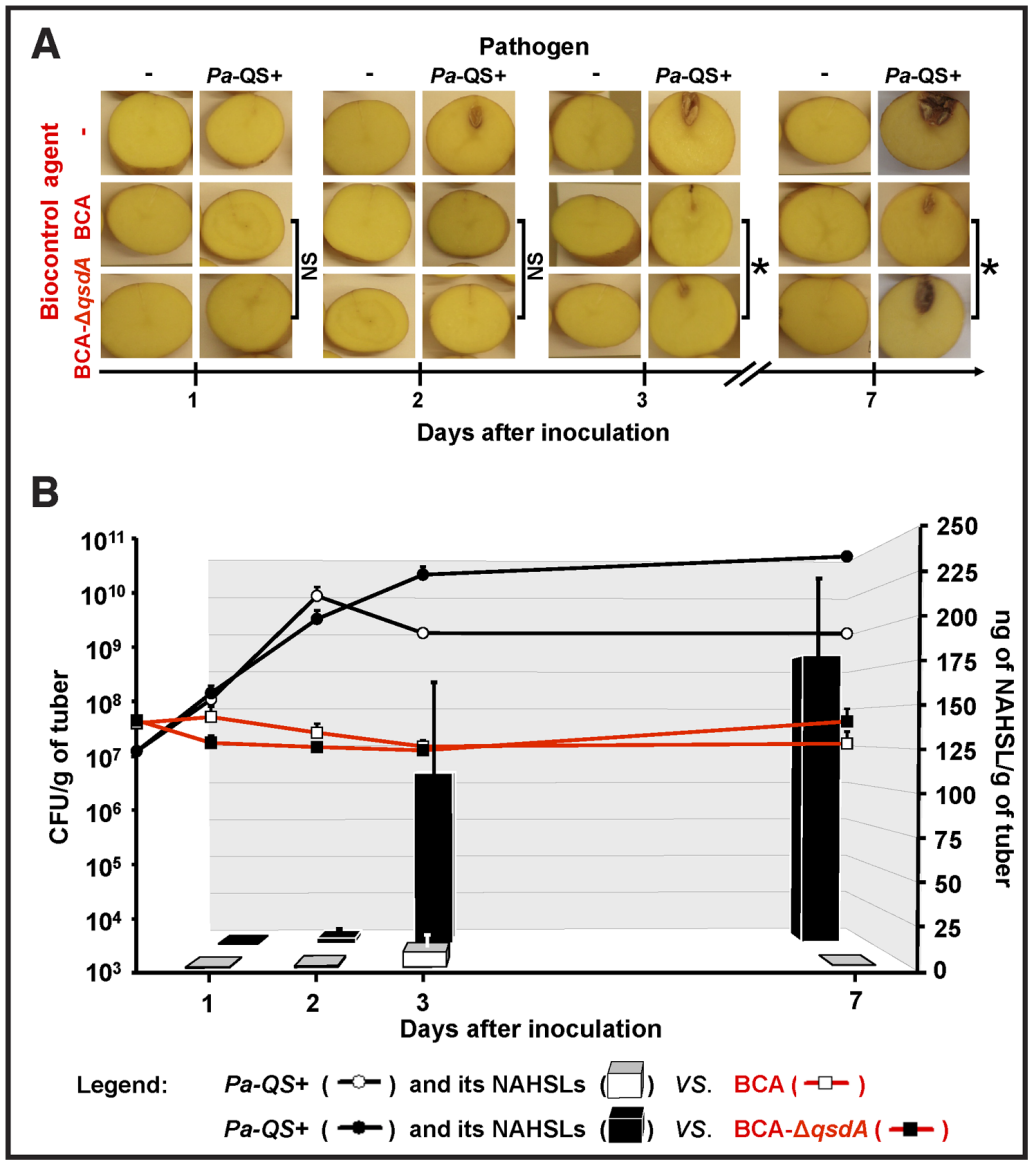
NAHSL-breakdown and biocontrol activity of the*R.erythropolis qsdA* deletion mutant in potato tubers. (A) The *R. erythropolis* R138 wild-type (BCA) and the *R. erythropolis* R138 Δ*qsdA* (BCA-Δ*qsdA*) strains were compared for biocontrol activity against *P. atrosepticum* 6276 (*Pa*-QS+) 1, 2, 3 and 7 days after inoculation of potato tubers. For the controls, one or both strains were replaced in the inoculum with a 0.9% NaCl solution. Significant differences (Mann and Whitney test; *α* = 0.05) in maceration symptoms between infected tubers inoculated with the BCA or the BCA-Δ*qsdA* are indicated with an asterisk. (B) Population dynamics of *P. atrosepticum* and *R. erythropolis* bacteria (CFU/g fresh weight of potato tubers; black and red lines respectively), and NAHSL concentrations (ng/g of potato tubers; black and white bars) were determined for each condition in potato tubers. For lines and bars, each value is the mean of three replicates with the standard deviation indicated. NS, non-significant; NAHSL, *N*-acyl homoserine lactone.

*P. atrosepticum* 6276 strains carrying or not carrying the empty vector pME6000 (*Pa*-QS+) were used both as typical NAHSL producers and virulent potato pathogens. These two strains produced similar amounts of NAHSL in the potato tubers (10 to 950 ng/g of tuber, 1 to 7 days after inoculation). *Pa*-QS+ induced tissue maceration, and the diameter of the lesion increased with time; the non-NAHSL producing derivative strain, *Pa*-QS**–** did not induce tissue maceration (Fig. 1A). This confirms that QS regulation is required for the virulence of *P. atrosepticum*. No maceration was detected in potato tubers inoculated only with *R. erythropolis* BCA, a bacterium that does not express the pectinolytic enzymes produced by soft-rot bacteria (Fig. 2A).

### *qsdA* Gene Transcription in *R. erythropolis* is Induced in Potato Tuber by *P. atrosepticum* NAHSLs

To analyze the *qsdA* gene transcription during biocontrol assays on potato tubers, the *R. erythropolis* R138 strain harboring a transcriptional *qsdA*::*gfp* fusion (BCA-*qsdA*::*gfp*) was generated (see the Materials and Methods). The extent of maceration was lower in tubers co-inoculated with *Pa*-QS+ and BCA-*qsdA*::*gfp* than in tubers inoculated with *Pa*-QS+ alone 2 to 7 days post-inoculation (*p*<0.05) (Fig. 1A). This indicates that the biocontrol capacity of *R. erythropolis* R138 had not been altered by transformation with the recombinant plasmid carrying the *qsdA*::*gfp* fusion.

CLSM of bacterial smears from inoculated tubers was used to assess GFP-associated fluorescence. No such fluorescence was detected in potato tubers inoculated with the BCA-*qsdA*::*gfp* strain alone (data not shown) or in combination with *Pa*-QS– (Fig. 1A). GFP-expressing bacteria were only detected between 2 and 7 days after co-inoculation with BCA-*qsdA*::*gfp* and *Pa*-QS+ (Fig. 1A), suggesting that the transcription of the *qsdA* gene is only induced if NAHSLs are produced. The population dynamics of the pathogen and of the BCA-*qsdA*::*gfp* strains were examined and inoculated tubers were assayed for NAHSLs. The density of the *Pa*-QS+ population in the presence of *R. erythropolis* increased from 10^7^ CFU/g to 6×10^9^ CFU/g 2 days post-inoculation (Fig. 1B). The population of *Pa*-QS– increased more slowly (from 10^7^ to 5×10^7^ CFU/g 3 days post-inoculation). In both cases, the populations remained relatively stable thereafter. The population of *R. erythropolis* remained constant until day 3 in the presence of *Pa*-QS+ and then decreased from 10^7^ to 3×10^6^ CFU/g. The concentration of NAHSL decreased 30 to 7,000 fold between days 1 to 7 in tubers co-inoculated with *Pa*-QS+ and BCA-*qsdA*::*gfp* strains, whereas there was no such decrease in tubers inoculated only with *Pa*-QS+ (data not shown). In contrast, in the presence of *Pa*-QS–, the population of *R. erythropolis* decreased more strongly with a similar fitness to that observed with the population inoculated alone (Fig. 1B). This suggests that the growth of BCA-*qsdA*::*gfp* is promoted by the assimilation of NAHSLs and/or tuber cell lysates associated with the presence of *Pa*-QS+. These various results clearly indicate that BCA-*qsdA*::*gfp* degrades NAHSL.

### QsdA Involvement in Quorum Quenching and Control of Tuber Soft-rot due to*P. atrosepticum*

To investigate the role of the γ-lactone catabolic pathway in the control of tuber soft-rot due to *P. atrosepticum*, a *qsdA* deletion mutant and a heterologous QsdA expression system were constructed (see Materials and Methods). The presence of a single copy of the *qsdA* gene in the genome of *R. erythropolis* R138 was verified by Southern hybridization using a *qsdA* probe (data not shown). The *R. erythropolis* R138 Δ*qsdA* (BCA-Δ*qsdA*) strain was compared to the *R. erythropolis* parental strain for its biocontrol ability against *P. atrosepticum* and for NAHSL-degrading activity. Tubers were co-inoculated with *P. atrosepticum Pa*-QS+ and each of the two *R. erythropolis* strains: significant differences in tissue maceration were observed from the third day (*p*<0.05) (Fig. 2A). The density of *P. atrosepticum* increased from 10^7^ to 9×10^9^ CFU/g by day 2 in the presence of the BCA strain and from 10^7^ to 2×10^10^ CFU/g by day 3 in the presence of the BCA*-*Δ*qsdA* strain (Fig 2B). The population of *P. atrosepticum* in the presence of the BCA strain decreased to 2×10^9^ CFU/g on day 3 and remained constant thereafter. In the presence of the BCA*-*Δ*qsdA* strain, the population of *P. atrosepticum* increased twofold after day 3. The density of *R. erythropolis* (both BCA and BCA*-*Δ*qsdA*) stayed relatively constant over the duration of the experiment at about 4×10^7^ CFU/g. NAHSL titers in tubers co-inoculated with the BCA*-*Δ*qsdA* strain were approximately 10 and 1,400 fold higher than in tubers co-inoculated with the parental strain on 3 and 7 days post-inoculation, respectively (Fig. 2B). This clearly implicates QsdA in NAHSL inactivation in potato tubers.

**Figure 3:**
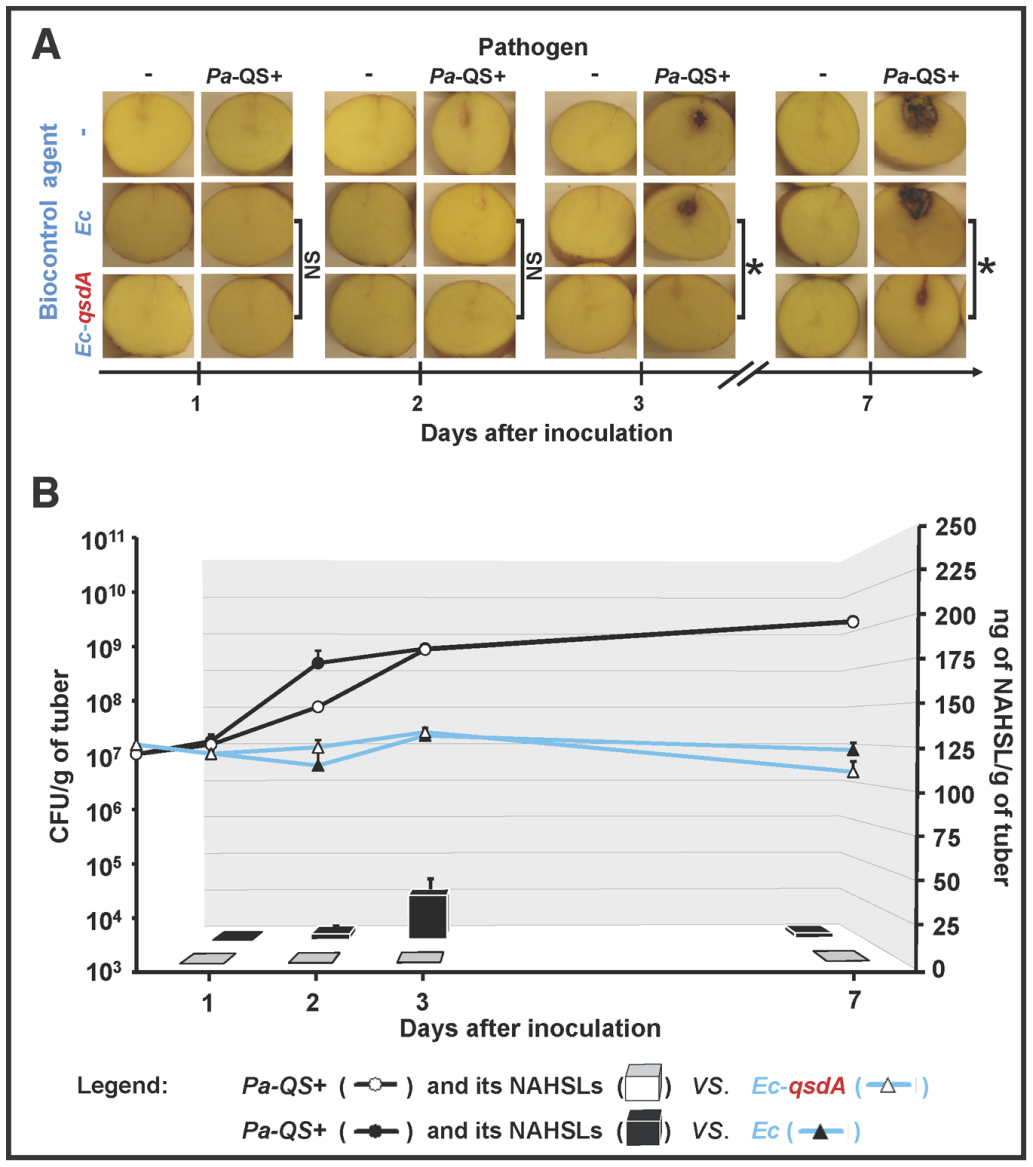
NAHSL-breakdown and biocontrol activity of the QsdA-expressing*E. coli* strain in potato tubers. (A) *E. coli* DH5α(pUC19) (*Ec*) and *E. coli* DH5α(pUC19-*qsdA*) (*Ec-qsdA*) were compared for biocontrol activity against *P. atrosepticum* 6276 (*Pa*-QS+) 1, 2, 3 and 7 days after inoculation of potato tubers. For the controls, one or both strains were replaced in the inoculum with a 0.9% NaCl solution. Significant differences (Mann and Whitney test; *α* = 0.05) in maceration symptoms between infected tubers inoculated with the *Ec* or the *Ec-qsdA* strains are indicated with an asterisk. (B) Population dynamics of *P. atrosepticum* and *E. coli* bacteria (CFU/g fresh weight of potato tubers; black and blue lines respectively), and NAHSL concentration (ng/g of potato tubers; black and white bars), were determined for each condition in potato tubers. For lines and bars, each value is the mean of three replicates with the standard deviation indicated. NS, non-significant; NAHSL, *N*-acyl homoserine lactone.

The *qsdA* gene was introduced into *E. coli*, a bacterium unable to degrade NAHSLs and the heterologous expression of QsdA was verified. The QsdA-expressing strain *E. coli* DH5α(pUC19-*qsdA*) (hereafter called *Ec-qsdA*) and the control *E. coli* DH5α strain carrying the empty pUC19 vector (*Ec*) were compared for biocontrol activity against *P. atrosepticum* and their ability to degrade NAHSL molecules. Potato tubers inoculated with the *P. atrosepticum* strain alone or in combination with *Ec* exhibited similar symptom severity (Fig. 3A); co-inoculation of tubers with the pathogen and *Ec-qsdA* resulted in a significantly less tissue maceration 3 and 7 days post-inoculation. The density of *P. atrosepticum* stayed constant for 24 h post-inoculation but then increased from 10^7^ to 10^8^ or 5×10^8^ CFU/g on day 2 in the presence of *Ec-qsdA* or *Ec*, respectively (Fig. 3B). A similar growth was noted from day 3 post-inoculation. The population density of *P. atrosepticum* increased to 2×10^9^ CFU/g on day 7 days whether co-inoculated with *Ec* or *Ec-qsdA*, whereas the *E. coli* population density (*Ec* or *Ec-qsdA*) remained constant over the seven-day duration of the experiment at about 10^7^ CFU/g. NAHSL was assayed in each condition: the amounts of these molecules in tubers co-inoculated with *Ec-qsdA* were approximately 20 to 100 fold lower than in tubers co-inoculated with *Ec* (Fig. 3B), confirming the *in planta* QS quenching ability of QsdA.

## Discussion

Rhodococcal cells have a large number of metabolic pathways and express numerous bioconversion and degradation activities. This has led to the use of these bacteria for the catabolism of recalcitrant molecules [34]–[36]. *R. erythropolis* both expresses an effective NAHSL-degrading activity and shows biocontrol ability to protect potato tubers; it is therefore an attractive candidate BCA against soft-rot bacteria by quorum quenching based-biocontrol [19], [37]. This strategy could be applied to the protection of numerous horticultural and vegetable crops and to the control of other plant pathogens that use *N*-3-oxo-octanoyl-L-HSL (for example *Agrobacterium* spp.) or γ-butyrolactones (*Streptomyces* spp.), as signaling molecules [38], [39].

The molecular mechanisms involved in this protection have not previously been identified *in planta*. Although the beneficial action of *R. erythropolis* has been evidenced in plant models, the activity responsible for the protection has mostly been studied using bacterial cells extracted from the host, then grown on synthetic media [13], [14], [19]. Recently, we discovered a catabolic pathway in *R. erythropolis* involved in the assimilation of various γ-lactones composed of a five-member ring linked to an aliphatic chain [21]. Here, we report an analysis of the involvement of this metabolic pathway in the biocontrol activity of *R. erythropolis* in potato tubers. We focused on the key marker of this catabolic route, the lactonase QsdA. QsdA catalyzes the ring-opening of γ-lactones, a selective and limiting operation involved in the first step of the pathway. Downstream from the *qsdA* gene in the *R. erythropolis* genome there are contiguous sequences encoding proteins strongly suspected to belong to the same pathway. For example, *fadD*, a gene under the same promoter as *qsdA*, codes for the long-chain fatty acid-CoA ligase FadD, which activates, by CoA thioester linkage, the aliphatic acids resulting from QsdA activity [21]. We first examined the transcriptional activity of the *qsdA* gene, in the form of a *qsdA::gfp* transcriptional fusion, in *R. erythropolis* co-inoculated with the pathogen into tubers. CLSM analysis revealed that GFP-expressing bacteria were first detectable 2 days after inoculation; consistent with this, NAHSL assays indicated that the *qsdA* gene was transcribed only in the presence of a sufficient concentration of NAHSL. No fluorescence was observed in the presence of the *P. atrosepticum* strain defective for NAHSL production. This threshold concentration of NAHSLs was reached when the *P. atrosepticum* population density reached a certain level, corresponding to the ‘quorum’ population density (after 48 h in our experimental model). This point in the potato infection marks the transition between the multiplication phase characterized by a rapid increase in the pathogenic bacterial density and the invasive phase typified by a stable population density and the massive production of lytic enzymes mediated by QS [40]. The absence of the characteristic multiplication and soft-rot phases in tubers inoculated with *Pa*-QS– was due to the absence of NAHSLs (or the production of amounts below the detection limits of the quantification methods). Indeed, NAHSL signals are essential for regulating the production of lytic enzymes, which release nutrients for the pathogen [8], [10]. Only the presence of *Pa*-QS+ promoted a sustainable survival of the populations of *R. erythropolis* in tubers over the seven-day duration of the experiment, enabling them to exert their biocontrol effect. An *E. coli* strain heterologously expressing QsdA was able to protect tubers against the pathogen with the same effectiveness as the wild-type *R. erythropolis* strain. This clearly confirms the role of QsdA in the protection of potato tubers against *P. atrosepticum*. Moreover, tissue maceration was significantly greater from the third day post-inoculation to the end of the experiment when the BCA-Δ*qsdA* mutant strain rather than the QsdA-expressing parental strain was used for co-infections. This difference was associated with a 10 fold greater *P. atrosepticum* population in the presence of the BCA-Δ*qsdA* strain and to higher NAHSL levels.

The deletion of the *qsdA* gene did not completely abolish the biocontrol activity of *R. erythropolis* R138. There are at least two possible explanations. Rhodococci produces multiple homologs of catabolic enzymes, enhancing metabolic versatility [35], [36]. Therefore, there may be one or more alternative enzymes for lactone catabolism working in addition to QsdA. The second possibility is that the inactivation of this pathway may provoke a switch in metabolism and the initiation of an alternative metabolic pathway taking over the function of the first pathway. This possibility deserves careful consideration, given that 3-oxo substituted-HSL molecules have been demonstrated to be bactericidal in Gram-positive bacteria [41]. Indeed, Gram-positive bacteria may have developed NAHSL-degrading enzymes to protect themselves against the antibacterial activity of NAHSLs and to enhance their survival in the natural environment [41], [42]. Were this the case, bacteria may possess alternative metabolic pathways for NAHSL inactivation to compensate for the inactivity of one of them. These alternative metabolic routes may involve a lactonase other than QsdA, an acylase which liberates a free homoserine lactone and a fatty acid, or an oxido-reductase whose activity results in silencing the QS-regulated processes; the degradation products of such activities cannot act as signal molecules [33]. Note that traces of these three activities have been detected *in vitro* in other *R. erythropolis* strains [43]–[45].

In conclusion, this work documents the nature of the signaling molecules used by *P. atrosepticum in planta* and their role in virulence. Previous *in vitro* assays show the intracellular production of catabolites (*N-*acyl homoserine) in *R. erythropolis* from NAHSLs by opening the lactone ring by QsdA, and also that other enzymes are required for the complete assimilation of lactones [21], [45]. Our work provides evidence for the involvement of this γ-lactone catabolic pathway in the breakdown of extracellular NAHSL and thus in the biocontrol activity of *R. erythropolis*. An important point for biocontrol applications is that this pathway is induced not by the invasion step or the presence of the pathogen, but by its QS-based communication. It would be valuable to characterize the mechanisms involved in signal transduction in the BCA, from NAHSL detection to the transcription of corresponding catabolic operons. The γ-lactone pathway of *R. erythropolis* is an example of a catabolic pathway in which the broad spectrum of the substrates allows it to contribute to bacterial nutrition [21], detoxification [41], [42] and the control of communication. Our experiments suggest that this pathway may well not be the only one involved in the control of soft-rot. These biocontrol pathways can be added to the list of those already described to protect plants from soft-rot bacteria, such as 2,4 diacetylphloroglucinol synthesis in *P. fluorescens* F113 [46] and the antagonism and/or the induction of systemic resistance by *Serratia plymuthica* A30 [47], [48]. More generally, the γ-lactone pathway differs from many of these other biocontrol mechanisms in that it is based on a catabolic principle; it is thus unlike antibiosis, iron competition or plant-induced systemic resistance based on the synthesis of secondary metabolic compounds.
